# PGC-1α promotes the survival of newborn neurons within AD hippocampus through activation of the FNDC5/BDNF/TrkB signaling pathway

**DOI:** 10.3389/fnmol.2025.1688694

**Published:** 2025-10-14

**Authors:** Yi-Jie Wang, Yu-Xin Wang, Cheng-Zhi Zou, Wei-Jun Zhang, Wen Pan, Jia-Qing Wang, Hua Wang, Xin Qian, Guo-Jia-Hao Han, Feng-Guo Liu, Jia Wang

**Affiliations:** ^1^The Fourth Affiliated Hospital of Jiangsu University, Zhenjiang, Jiangsu, China; ^2^Department of Laboratory Medicine, School of Medicine, Jiangsu University, Zhenjiang, Jiangsu, China

**Keywords:** PGC-1α, Alzheimer’s disease, newborn neurons, BDNF, survival

## Abstract

**Background:**

The learning and memory impairments observed in Alzheimer’s disease (AD) are strongly associated with impaired neurogenesis in the hippocampal region. Our previous research has highlighted the potential of peroxisome proliferator-activated receptor gamma coactivator-1 alpha (PGC-1α) in ameliorating AD-related pathological changes. As a key metabolic regulator, PGC-1α is highly expressed in energy-demanding tissues such as the hippocampus. However, its specific roles and underlying mechanisms in AD-associated neurogenesis remains largely unclear.

**Objective:**

This study aimed to elucidate the precise role and molecular mechanisms by which PGC-1α regulates the survival of newly generated neurons during neurogenesis in the AD-affected hippocampus.

**Methods:**

Using combined models of PGC-1α overexpression in the hippocampal dentate gyrus (DG) of AD-model mice and PGC-1α knockout mice, we investigated the effects of gain- and loss-of-function of PGC-1α on the regulation of the FNDC5/BDNF/TrkB signaling pathway, as well as on the survival of newborn neurons in the AD-affected hippocampus.

**Results:**

Our findings demonstrate that PGC-1α enhances the survival of newly generated neurons in the AD-affected hippocampus. Furthermore, PGC-1α functions acts as an upstream regulator of the FNDC5/BDNF/TrkB signaling pathway, and its knockdown suppresses neuronal survival by inhibiting this pathway.

**Conclusion:**

These results indicate that PGC-1α serves as a critical mediator in the FNDC5/BDNF/TrkB signaling pathway within newborn neurons. Enhancing PGC-1α expression, either pharmacologically or through alternative approaches, may therefore represent a promising therapeutic strategy for Alzheimer’s disease.

## 1 Introduction

In specific brain areas, adult neurogenesis refers to the lifelong generation of functional neurons from neural precursors (e.g., the dentate gyrus [DG]) (Fares et al., 2019). This process encompasses the complete developmental trajectory of neuronal maturation. Within the DG, actively proliferating neural precursor cells (NPCs) differentiate into neuroblasts. These neuroblasts subsequently mature into immature neurons (Pan et al., 2025), which migrate to the inner granule cell layer and eventually develop into fully mature granule cells within the hippocampal formation (Lattanzi et al., 2020). Newly formed neurons exhibit dendritic growth into the molecular layer and axonal extension through the hilus to CA3, enabling their integration into functional neural networks (Dong et al., 2019).

A substantial amount of new cells are generated daily in the dentate gyrus of typical adult brains, with estimates reaching up to 9,000 in young adult rats (Cameron and McKay, 2001). Longitudinal monitoring over months revealed a significant decline (30%–70%) in the numbers of progenitor cells and young neurons within BrdU-labeled cohorts (Winner et al., 2002). Although the exact age and identity of the dying cells have not been examined, it is probable that a substantial proportion of newly generated cells undergo apoptosis prior to reaching maturity (Wang et al., 2022b). Research has demonstrated that newly born cells experience two key phases for survival and maturation: the first occurs during the intermediate progenitor and neuroblast stages, while the second takes place as immature neurons integrate into neural circuits (Ming and Song, 2011). Each stage is associated with specific developmental phases and neuronal cell types. During the early survival critical period (1–2 weeks post-mitosis), neuroblasts and immature neurons exhibit doublecortin (DCX) expression, while during the late integration critical period (3–6 weeks post-mitosis), functionally integrated dentate granule cells (DGCs) express neuronal nuclear antigen (NeuN) (Zhao et al., 2018).

Recent studies have shown dysregulation in adult hippocampal neurogenesis in both AD patients and mouse models (Pan et al., 2025). Notably, several key molecules implicated in AD, such as presenilin 1 (PS1), amyloid precursor protein (APP), and their metabolites, play critical roles in regulating the generation of new hippocampal neurons (Mu and Gage, 2011). Furthermore, it is imperative to investigate which signaling pathways support the survival of newborn neurons and whether enhancing their survival could alleviate impaired neurogenesis for AD treatment. Identifying potential therapeutic targets that promote neurogenesis and ensure the survival of postmitotic neurons has emerged as a central focus of our research interest.

Peroxisome proliferator-activated receptor gamma coactivator-1 alpha (PGC-1α), a key transcriptional coactivator, is abundant in tissues relying on oxidative metabolism for ATP production, including the brain. Our prior studies demonstrated that PGC-1α overexpression in cortical neurons and brain tissue improves AD-like behavioral deficits, such as impaired spatial memory, working memory, and sensorimotor gating (Wang et al., 2022a). Additionally, adeno-associated virus (AAV)-mediated PGC-1α delivery to the lateral parietal association (LPtA) cortex reduces AD-related neuronal apoptosis (Shi et al., 2024). However, the role of PGC-1α in adult hippocampal neurogenesis remains unclear, despite its significant downregulation in the hippocampal DG during AD progression (Wang et al., 2025).

In the course of development, programmed cell death–commonly known as apoptosis–serves a vital function in refining the alignment between neuronal populations and available synaptic targets. This process efficiently removes neurons that fail to form proper connections or receive sufficient trophic support. Many studies have identified survival-promoting signals in adult neurogenesis, including neurotrophic factors, hormones, and extracellular signaling molecules (Faigle and Song, 2013). These pro-survival effects inhibit apoptosis-inducing pathways. In adult neurogenesis, brain-derived neurotrophic factor (BDNF) regulates the differentiation and survival of newborn hippocampal neurons (Chan et al., 2008).

Given the well-established interplay between PGC-1α and BDNF in maintaining brain health through exercise (Bi et al., 2024), as well as the significant inhibition of neuronal apoptosis by AAV-PGC-1α infusion in AD brains (Wang et al., 2022a), we propose that PGC-1α might serve as a key regulator in the BDNF-dependent survival of adult hippocampal neurons during AD. By utilizing the APP/PS1 transgenic model for AD combined with AAV-PGC-1α infusion, we achieved strong overexpression of PGC-1α specifically within the DG region of the hippocampus in these mice. Through activation of the FNDC5-BDNF axis, we confirm that PGC-1α promotes both short- and long-term survival of newborn DG neurons. These combined effects result in the generation of postmitotic neurons within the hippocampus in AD animals. Furthermore, using gene targeting strategies, we developed a murine model with conditional *Pgc-1*α ablation in Calb1^+^neurons by crossing Calb1-Cre knock-in mice with PGC-1α^fl/fl^ mice. Significant suppression of fibronectin type III domain-containing protein 5 (FNDC5) and BDNF expression was observed following PGC-1α downregulation in the hippocampus. Consequently, our findings indicate that PGC-1α serves as an upstream regulator of the FNDC5-BDNF signaling pathway, which is implicated in neurogenesis within the hippocampus.

## 2 Materials and methods

### 2.1 Animals

C57BL/6 mice and other genotypes were kept under controlled conditions: 12-h light/dark cycle, free access to water and food, and 22 ± 2° C. All animal experiments followed the guidelines of Jiangsu University, the Chinese Council on Animal Care, and the NIH Guide (NIH Pub. No. 80-23, revised 1978).

#### 2.1.1 Generation of Pgc-1α conditional knockout mice

B6N.129 (FVB)-*Ppargc1*α^tm2.1Brsp/J^ (PGC-1α^fl/fl^) mice were procured from the Jackson Laboratory (Catalog No. 009666). B6/JGpt-Calbindin (Calb1) *^em1Cin(P2A–iCre)^*/Gpt (Calb1-Cre) mice were obtained from Jiangsu Gem Pharmatech Co., Ltd., China (No. T006202). The Calb1-Cre::PGC-1α^fl/fl^ (*Pgc-1*α CKO) mice were generated by crossing Calb1-Cre mice with PGC-1α^fl/fl^ mice. Calb1-Cre::PGC-1α^+/+^(Calb1-Cre) mice were used as controls.

The genotypes of the PGC-1α^fl/fl^ offspring were identified via PCR analysis using specific primers (5′-TCC AGT AGG CAG AGA TTT ATG AC-3′, and 5′-TGT CTG GTT TGA CAA TCT GCT AGG TC-3′) to amplify fragments of 400 bp and 360 bp, respectively. The genotypes of the Calb1-Cre offspring were determined by PCR analysis with primers (5′-CTT AAT GGA CTG GTG TAG CAA GCA GT-3′, and 5′-CTG CAC ACA GAC AGG AGC ATC TTC-3′), amplifying a fragment of 444 bp in length (Figure 4A).

#### 2.1.2 Forced expression of PGC-1a in the hippocampal DG of APP/PS1 brain

**FIGURE 1:**
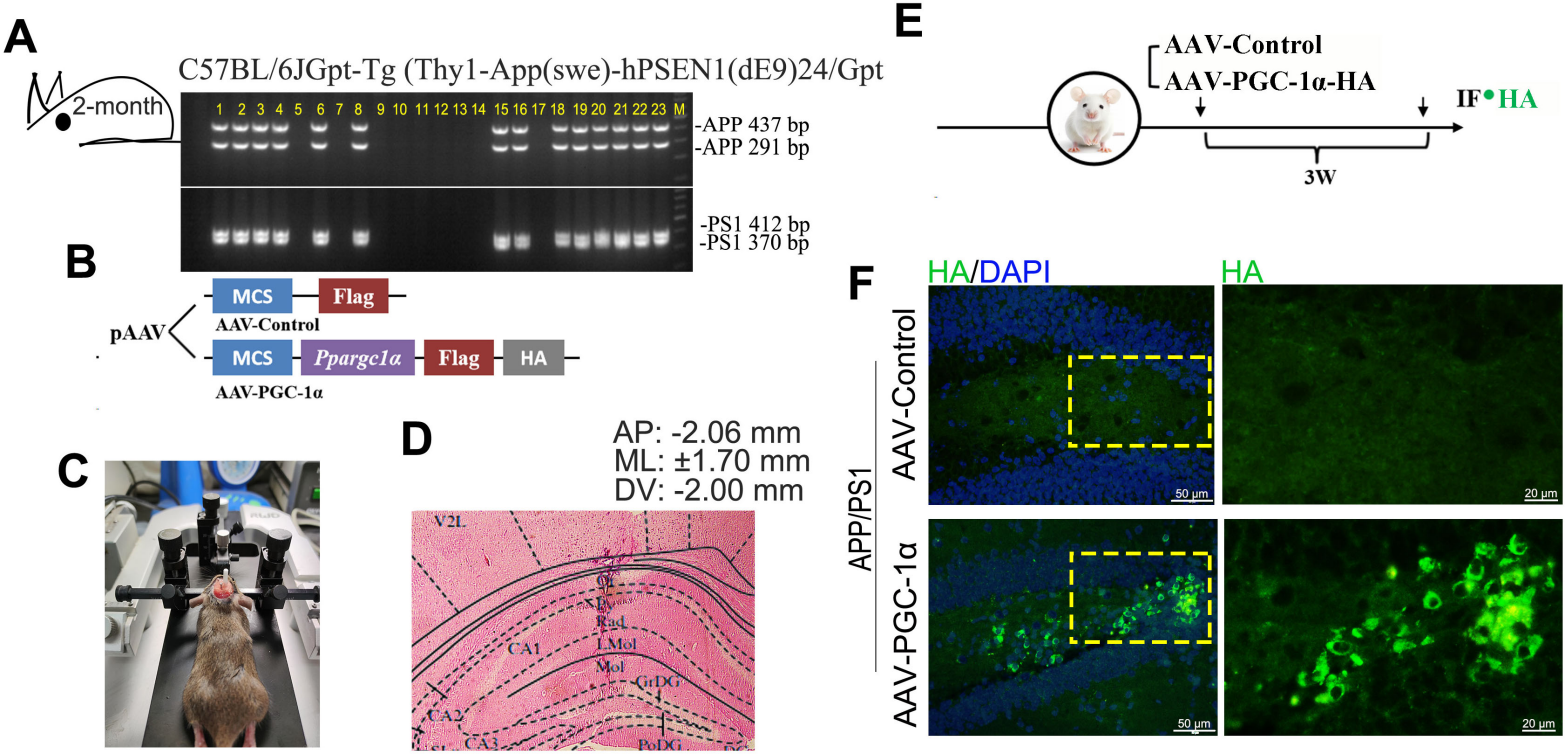
PGC-1α is overexpressed in the hippocampal DG of AD brain. **(A)** APP/PS1 offspring genotypes were determined by PCR using specific primers for APP and PS1. The 437 and 291 bp bands correspond to APP alleles, while the 412 and 370 bp bands correspond to PS1 alleles. Lanes 1–4, 6, 8, 15–16, and 18–23 indicate APP/PS1 genotype individuals. **(B,C)** PGC-1α overexpression was induced in APP/PS1 mouse hippocampus via microinjection of AAV-PGC-1α (AAV-CMV-*Ppargc1*α-m-FLAG-HA) or AAV-Control. **(D)** Eosin-stained coronal brain section showing infusion site placement in the hippocampus. **(E,F)** PGC-1α expression patterns were assessed with an anti-HA antibody 21 days post-injection. Green, HA-labeled PGC-1α; Blue, DAPI.

The heterozygous APP/PS1 double transgenic mice (2 × Tg-AD) [C57BL/6JGpt-Tg (Thy1-APP (swe)-hPSEN1 (dE9)24/Gpt], which were maintained on C57BL/6 background, were acquired from GemPharmatech (Nanjing, China; Catalog No. T053302). Offspring genotypes were confirmed by PCR using specific primers. For APP amplification: 5′-AAG TAA TGA AGT CAC CCA GCA GG-3′, 5′-CGA GGA AAC TGA TCC TCT AGG TG-3′ and 5′-GGG TTG ACA AAT ATC AAG ACG GAG-3′, 5′-CGT TTC TCC CCA TGT TCT GAG A-3′ (437 bp and 291 bp products). For PS1 amplification: 5′-AAG TAA TGA AGT CAC CCA GCA GG-3′, 5′-CGT ACA GTA TTG CTC AGG TGG TTG-3′ and 5′-AGG AAC TTT CCA GCA GTA TCC TC-3′, 5′-CGT TTC TCC CCA TGT TCT GAG A-3′ (412 bp and 370 bp products) (Figure 1A).

The APP/PS1 transgenic mouse was administered a ketamine/xylazine mixture (73/11.6 mg/kg) via intraperitoneal injection, then secured in a stereotaxic frame (RWD) for the procedure. A glass pipette was placed within the hippocampus with the following coordinates relative to bregma: Anteroposterior (AP): −2.06 mm, Mediolateral (ML): ± 1.70 mm, and Dorsoventral (DV): −2.00 mm (Figure 1D). A viral vector suspension (0.5 μL), containing 2 × 10^12^ viral genome copies (vgc) of AAV2-CMV-*Ppargc1*α-m-FLAG-HA (AAV-PGC-1α) and 2 × 10^12^ vgc of AAV2-CMV-FLAG-control (AAV-Control), was microinjected into the target region using a Nanoliter 2000 injector (World Precision Instruments) (Figure 1B). The plasmids were provided by Applied Biological Material Co. (abm, Zhenjiang, China). AAV-mediated protein expression peaks 3 weeks post-infusion and remains stable thereafter (Shi et al., 2024; Wang et al., 2022b). Molecular analyses were conducted 3 weeks after infusion (Figure 1E), and virus was detected by immunofluorescence staining (Figure 1F).

### 2.2 Materials

Unless otherwise specified, all tissue culture media were supplied by thermofisher (Carlsbad, CA), and all chemicals and reagents were provided by MilliporeSigma (St. Louis, MO). Antibodies used in this study were as follows: PGC-1α (Bioss, cat # bsm-1832R, Beijing, China, 1: 500), Flag (abm, cat # G188, Zhenjiang, China, 1: 600), HA (Bioss, cat # bsm-33003M, Beijing, China, 1: 600), rabbit anti-NeuN (Wanleibio, cat # WL03099, Shenyang, China, 1: 100), mouse anti-NeuN (Proteintech, cat # 66836-1-lg, Wuhan, China, 1: 600), DCX (Proteintech, cat # 13925-1-AP, Wuhan, China, 1: 600), BDNF (Wanleibio, cat # WL0168, Shenyang, China, 1: 1,000), FNDC5 (ABmart, cat # PK57698, Shanghai, China, 1: 1,000), TrkB (MedChem Express, cat #HY-P80923, Shanghai, China, 1: 800), β-Tubulin (abm, cat # G098, Zhenjiang, China, 1: 1,000). HRP-conjugated secondary antibodies were purchased from Beyotime Biotechnology (goat anti mouse cat # A0216 and goat anti rabbit cat # A0208, Shanghai, China, 1: 1,000). Immunohistochemistry secondary antibodies were purchased from Boster Biological Technology co (DyLight 594- and 488-labeled conjugated AffiniPure Goat Anti-Rabbit IgG, 1: 250; DyLight 594- and 488-labeled conjugated AffiniPure Goat Anti-Mouse IgG, 1: 250).

### 2.3 Eosin staining assay

Mice were anesthetized and perfused transcardially with PBS followed by 4% paraformaldehyde (PFA). Brains were fixed in 4% PFA for 48 h and embedded in paraffin. Sections were washed with PBS, stained with eosin after brief color separation using 1% hydrochloric acid alcohol (75% ethanol), dehydrated with graded ethanol (95 and 100%, 5 min each), and cleared with dimethylbenzene (10 min, two changes). Coverslips were mounted (Dong et al., 2019). The placement of injector tips was confirmed using Paxinos and Watson’s atlas (Figure 1D). Data from infusion sites outside the target area were excluded from statistical analyses.

### 2.4 Immunohistochemistry

Mice were anesthetized and perfused transcardially with PBS, followed by 4% PFA in 0.01 M PBS (pH 7.4). Brain tissue was fixed in 4% PFA for 48 h, dehydrated through graded ethanol, and embedded in paraffin. Coronal hippocampal sections (6 μm) were prepared, dewaxed with xylene, rehydrated, and subjected to antigen retrieval. Immunofluorescence was performed using primary antibodies. The following day, samples were washed with PBS, incubated with fluorescent secondary antibodies in the dark, and stained with DAPI for nuclear visualization (Wang et al., 2022c). Sections were mounted on gelatin-coated slides for analysis.

### 2.5 EdU labeling and detection

To investigate the impact of PGC-1α on the short- and long-term survival of newborn neurons, mice received a single intraperitoneal injection of EdU (Beyotime, catalog # ST067, Shanghai, China). Survival periods were set at 2 or 6 weeks after the peak of AAV-mediated transgenic protein expression at 3 weeks post-injection. For EdU staining, tissues were processed using the BeyoClick EDU-488 Kit (C0071S, Beyotime). Samples were incubated with primary antibodies against DCX (immature neurons) or NeuN (mature neurons) after PBS washing. Nuclei were stained with DAPI, and sections were mounted onto Superfrost slides (Fisher Scientific, 12-550-15).

### 2.6 Image acquisition and analyses

For cell counting, 6 μm coronal hippocampal sections were analyzed in 3–5 representative sections per brain by an experimenter blinded to the genotype. Images were captured using an Olympus BX41 epifluorescent microscope with 20 × or 40 × objectives. Cells were quantified in at least three sections per brain, and data were collected from at least six brains per genotype. The DG region (hilus, granule cell layer, and molecular layer) was outlined for counting using ImageJ 2.0.0. Figures were assembled in Photoshop CC.

### 2.7 RT-qPCR

Total RNA was extracted from mouse hippocampal tissues using the Cell/Tissue Total RNA Kit (beyotime, Shanghai, China). The RNA was reverse-transcribed into cDNA using the Hifair^®^ III 1st Strand cDNA Synthesis SuperMix for qPCR (Yeasen). Two-step RT-qPCR gene expression analysis was performed with the SYBR Green Assay Kit (Vazyme Biotech). Primer sequences are listed in Table 1. GAPDH served as the internal control for mRNA normalization, and all reactions were conducted in sextuplicate.

**TABLE 1:** Primers used for RT-qPCR analyses.

Gene of Interest	Primer orientation	Primer sequence
*Fndc5*	Forward	5′-TTGCCATCTCTCAGCAGAAGA-3′
	Reverse	5′-GGCCTGCACATGGACGATA-3′
*BDNF*	Forward	5′-TCATACTTCGGTTGCATGAAGG -3′
	Reverse	5′-AGACCTCTCGAACCTGCCC-3′
*TrkB*	Forward	5′-CTGGGGCTTATGCCTGCTG -3′
	Reverse	5′-AGGCTCAGTACACCAAATCCTA-3′
*GAPDH*	Forward	5′-GGTGAAGGTCGGTGTGAACG-3′
	Reverse	5′-CTCGCTCCTGGAAGATGGTG-3′

### 2.8 Cell lines, plasmids and transfection

Neuro-2a (N_2_A) neuroblastoma cells (Shanghai Academy of Sciences, TCM29) were transfected with Lipofectamine 2000 (Life Technologies, SCSP-502) at 30%–50% confluency. For co-transfection experiments, plasmids were used at a 2 μg: 2 μg ratio. The expression plasmid pCAX-APPSwe/Ind (APPSwe) was constructed by inserting the gene encoding human APP 695 with the Swedish/Indiana mutation into the pCAX Vector backbone. APPSwe plsmid, pEnCMV-*Pargc1a*-Flag (*Pgc-1α*), and the control plasmid pEnCMV were obtained from Miaoling Bio (Wuhan).

### 2.9 Cell immunofluorescence staining

N_2_A cells were seeded on glass coverslips in 35-mm culture plates and cultured at 37° C with 5% CO2 until they achieved 50% confluence. The cells were then fixed with 4% paraformaldehyde (Thermofisher) for 20 min at room temperature, permeabilized using 0.3% Triton X-100 in PBS for 30 min, and blocked for 1.5 h. Following this, the coverslips were incubated overnight at 4° C with primary antibodies against BDNF and NeuN, and subsequently incubated with secondary antibodies for 1 h at room temperature. After washing, the coverslips were placed in a fluorescence-preserving mounting medium (Invitrogen) and sealed with clear nail polish.

### 2.10 Western blot analyses

Tissue samples or cells were lysed, and Western blot analyses were conducted following previously reported methods (Wang et al., 2021). Typically, 50 μg of protein was loaded per lane. Protein visualization was achieved using an enhanced chemiluminescence system (ECL, Meilunbio, catalog number MA0186, Shanghai, China). The signal intensity was quantified through densitometric analysis.

### 2.11 Statistical analyses

Normality was assessed for all continuous variables using the Shapiro-Wilk test with a significance level of α = 0.05. All datasets met the assumptions of normality as assessed by the Shapiro-Wilk test; no data were excluded on this basis (Supplementary Table 1). Statistical analyses were performed using StatView 5.01 (Abacus Concepts, Inc., Berkeley, CA, 1992). Unpaired two-tailed Student’s *t*-tests were used for imaging and immunoblotting data. Sample sizes are indicated in figure legends, with significance defined as *p* < 0.05. Values are expressed as means ± S.E.M.

## 3 Results

### 3.1 AAV-PGC-1α infusion induced overexpression of PGC-1α in the dentate gyrus (DG) of the hippocampus in AD brains

To investigate the role of PGC-1α in regulating neuron population and its potential mechanisms, we induced PGC-1α overexpression in the hippocampal DG of 2 × Tg-AD mice by microinjecting AAV-PGC-1α (AAV-CMV-*Ppargc1*α-m-FLAG-HA) or AAV-Control bilaterally (Figures 1A–D). Robust PGC-1α expression with the reporter HA was observed 21 days post-injection (Figures 1E, F).

### 3.2 PGC-1α promotes the survival of newborn granule neurons in AD

**FIGURE 2:**
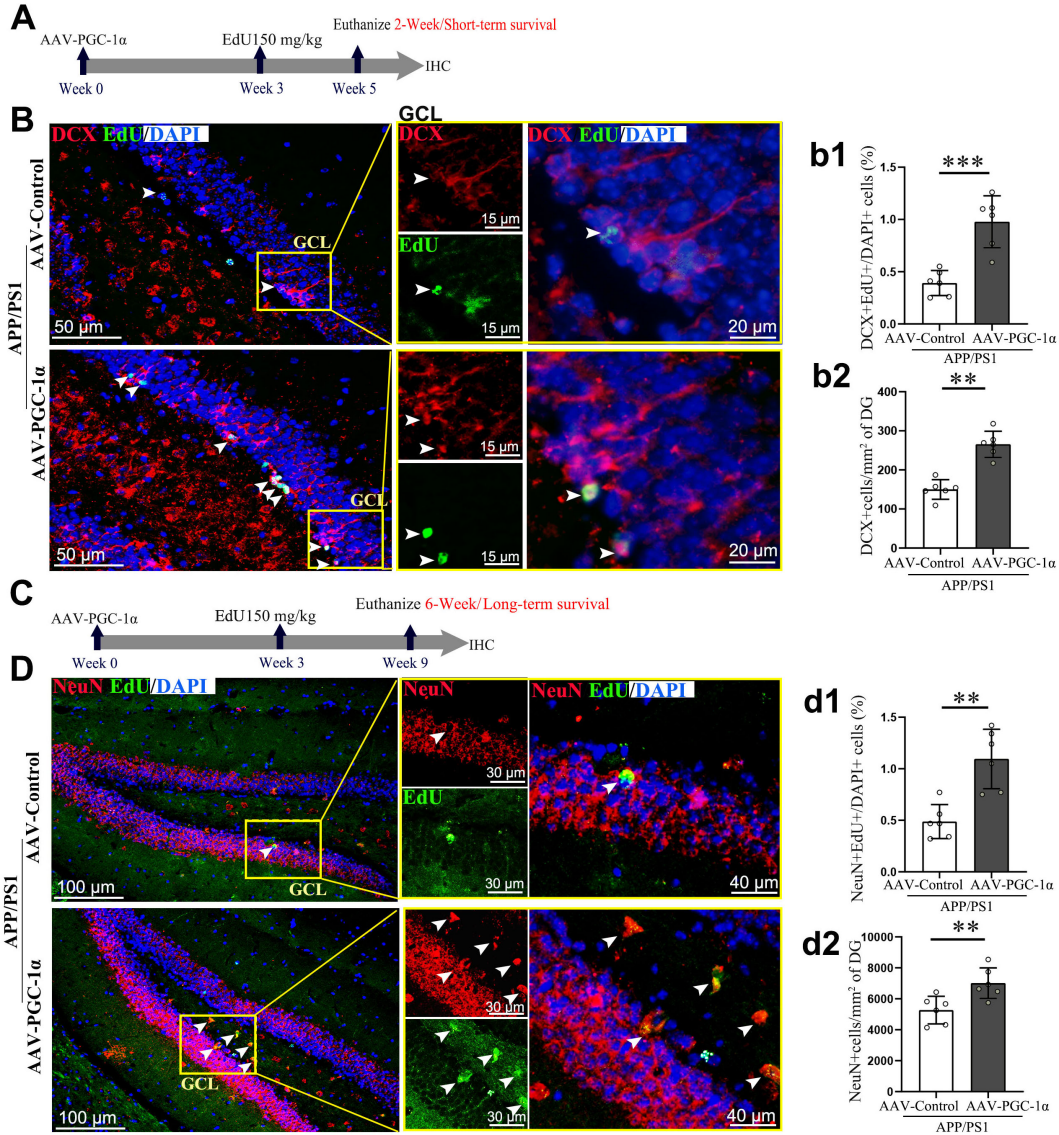
PGC-1α promotes the differentiation and survival of newborn neurons in the DG of AD hippocampus. **(A)** Three weeks after the stereotaxic injection of the AAV-Control or AAV-PGC-1α into the APP/PS1 mice, EdU was administered intraperitoneally. Two weeks later, **(B)** hippocampal sections were immunolabeled with EdU (Green), DCX (Red), and DAPI (Blue). GCL, granule cell layer. **(b1)** Short-term survival of immature neurons is quantified as EdU^+^; DCX^+^/DAPI^+^ cells (%). **(b2)** DCX protein density (cells/mm^2^) was measured in the DG. **(C)** Three weeks after AAV injection, EdU was administered. Six weeks later, **(D)** hippocampal sections were immunolabeled with EdU (Green), NeuN (Red), and DAPI (Blue). **(d1)** Long-term survival of mature neurons is quantified as EdU+; NeuN^+^/DAPI^+^ cells (%). **(d2)** NeuN protein density (cells/mm^2^) was measured in the DG. Values are means ± S.E.M., *n* = 6/group. Fisher’s LSD test: **p < 0.01, ***p < 0.001 for comparisons between AAV-Control and AAV-PGC-1α groups.

To investigate the impact of PGC-1α on short- and long-term survival of adult-born neurons in the DG, we compared EdU-labeled immature (DCX^+^) and mature (NeuN^+^) neurons in AAV-Control and AAV-PGC-1α-infused AD mice. After a 2-week EdU pulse-labeling period (Figure 2A), immature neuron survival was higher in AAV-PGC-1α-infused mice (Figures 2B, b1). After a 6-week EdU pulse-labeling period (Figures 2C), mature neuron survival was also increased (Figures 2D, d1). Quantitative analyses confirmed upregulated numbers of DCX^+^ immature neurons (Figures 2B, b2) and NeuN^+^ mature neurons (Figures 2D, d2) in PGC-1α-overexpressing hippocampal tissues during AD progression.

### 3.3 PGC-1α expands the pool of neurons within AD hippocampus by regulating FNDC5/BDNF/TrkB pathway

**FIGURE 3:**
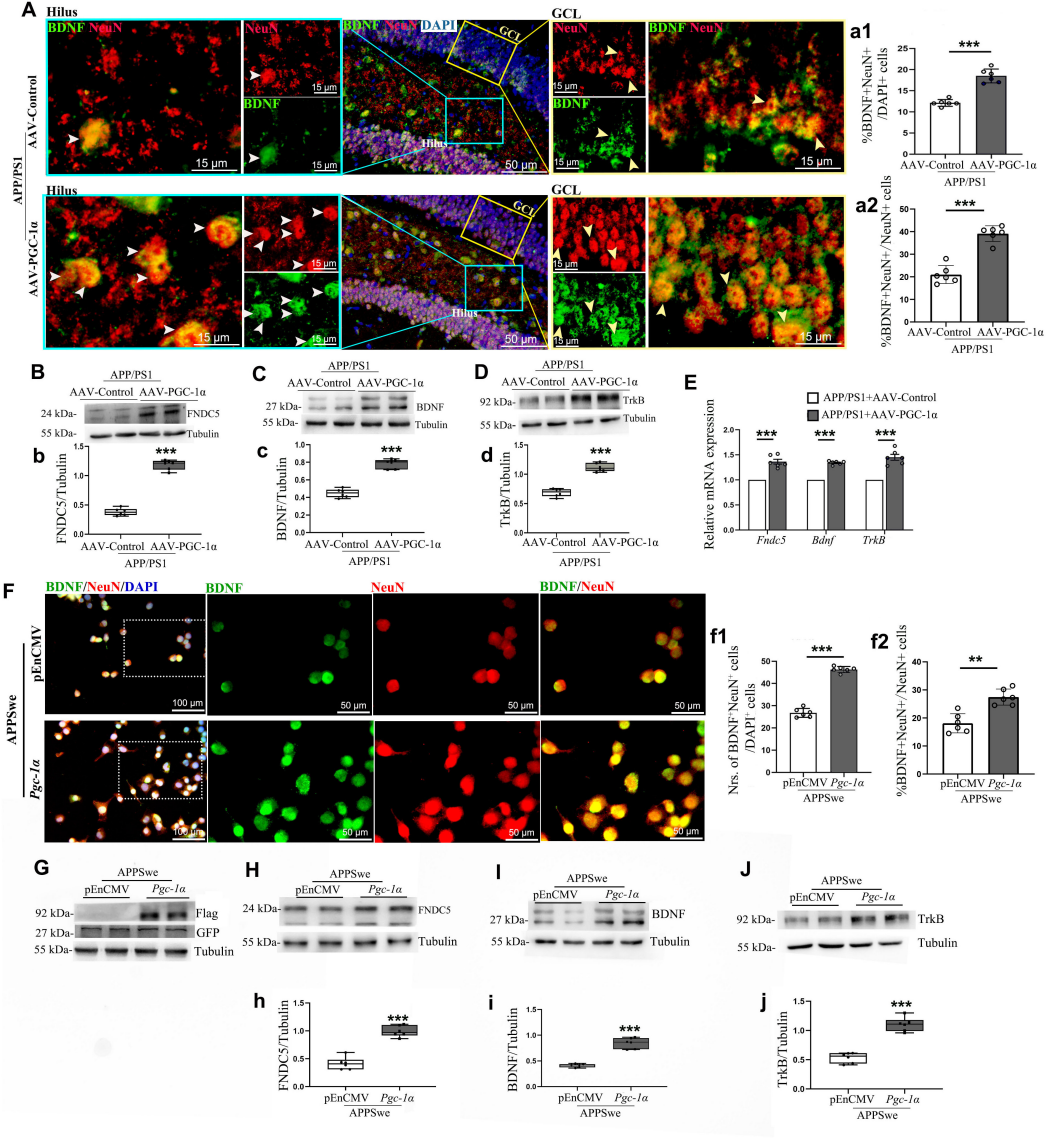
PGC-1α promotes newborn neuron proliferation in the AD hippocampus via the FNDC5/BDNF/TrkB pathway. AAV-Control or AAV-PGC-1α was microinjected bilaterally into the DG of 2 × Tg-AD mice. **(A)** Immunolabeling with BDNF (Green), NeuN (Red), and DAPI (Blue) was performed on hippocampal sections. GCL, granule cell layer. White arrowhead: BDNF^+^; NeuN^+^ cells in the hilus of the AD hippocampus; Yellow arrowhead: BDNF^+^; NeuN^+^ cells in the GCL of the AD hippocampus. The quantification of numbers of BDNF^+^; NeuN^+^ cells in AD hippocampus was presented as **(a1)** %BDNF^+^; NeuN^+^ /DAPI^+^ cells and as **(a2)** %BDNF^+^; NeuN^+^ /NeuN^+^ cells. *n* = 6/group. Hippocampal lysates were analyzed by immunoblotting for **(B,b)** FNDC5, **(C,c)** BDNF, and **(D,d)** TrkB, with Tubulin as a loading control. **(E)** RT-qPCR assessed mRNA levels of *Fndc5*, *Bdnf*, and *TrkB* under both conditions. N_2_A cells were transfected with pEnCMV/*Pgc-1*α and APPSwe plasmids for 24 h. **(F)** Confocal images show NeuN [Red]/BDNF [Green] colocalization in co-transfected cells. The quantification of numbers of BDNF^+^; NeuN^+^ cells in co-transgenic cells was presented as **(f1)** % BDNF^+^; NeuN^+^/DAPI^+^ cells and as **(f2)** % BDNF^+^; NeuN^+^/Neun^+^ cells. *n* = 6/group. **(G)** N_2_A cells were transfected with the pEnCMV/*Pgc-1*α plasmid and a plasmid-encoding APPSwe for a period of 24 h. Western blot analyses confirmed the overexpression of GFP-labeled APPSwe and Flag-labeled PGC-1α in the transfected cells. Cell lysates were immunoblotted for **(H,h)** FNDC5, **(I,i)** BDNF, and **(J,j)** TrkB, with Tubulin as a loading control. Values are means ± S.E.M., *n* = 6/group, and significant levels set at **p < 0.01, ***p < 0.001 indicating differences between AAV-Control and AAV-PGC-1α-infused AD mice, or APPSwe + pEnCMV and APPSwe + *Pgc-1*α groups.

Granule neurons (GNs) are the terminal stage of differentiation and express markers such as NeuN, Prox-1, Calb1, and β III-tubulin (Dong et al., 2019). Our prior findings showed that PGC-1α overexpression increases mature neuron populations in the AD hippocampus (Wang et al., 2025). However, the underlying mechanisms remain unclear. To address this gap, we co-labeled BDNF with NeuN and found that PGC-1α enhances BDNF^+^; NeuN^+^ cell populations (Figures 3A, a). To further elucidate these findings, we transfected N_2_A cells with pEnCMV/*Pgc-1*α and APPSwe plasmids for 24 h. Results confirmed a significant increase in BDNF^+^; NeuN^+^ neurons upon *Pgc-1*α transfection (Figures 3F, f).

Given the established role of the PGC-1α/ERR transcription complex in protecting neurons through activation of the FNDC5/BDNF signaling pathway (Cameron and McKay, 2001), we investigated how PGC-1α regulates neuronal differentiation and maturation in AD using *in vivo* and *in vitro* models. Results showed that PGC-1α enhances FNDC5, BDNF, and TrkB expression (Figures 3B–D, b–d). RT-qPCR confirmed upregulated *Fndc5*, *Bdnf*, and *TrkB* transcription in PGC-1α-overexpressing hippocampal tissues during AD progression (Figure 3E). To validate the FNDC5/BDNF/TrkB pathway’s contribution to PGC-1α-mediated effects, N_2_A cells were transfected with GFP-tagged APPs we plasmid and either pEnCMV or *Pgc-1a* (Figure 3G). Western blot analyses revealed increased FNDC5, BDNF, and TrkB levels in *Pgc-1a*-transfected AD cells (Figures 3H–J, h–j).

### 3.4 Pgc-1α conditional knockout significantly reduces PGC-1α expression in the hippocampus

**FIGURE 4:**
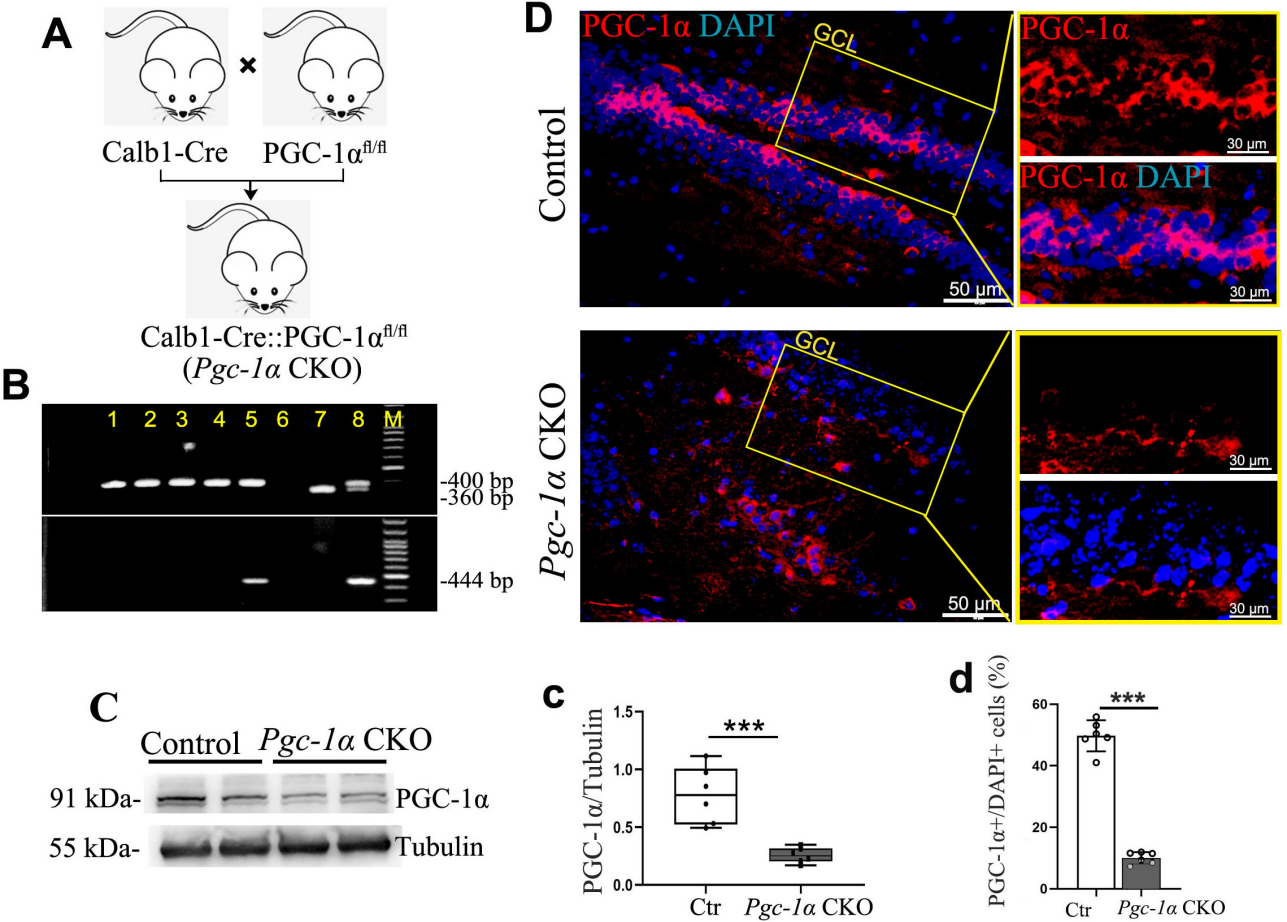
Generation of PGC-1α conditional knockout (CKO) mice and confirmation of reduced hippocampal PGC-1α expression. **(A)** The Calb1-Cre::PGC-1α^fl/fl^ (*Pgc-1*α CKO) mice were generated by crossing Calb1-Cre mice with PGC-1α^fl/fl^ mice. **(B)** PCR products from genomic DNA of *Pgc-1*α CKO. The 400- and 360-bp bands resulted from the amplification of *Pgc-1*α alleles, and 444-bp band resulted from the amplification of Calb1-Cre allele. **(C,c)** Hippocampal lysates were immunoblotted with anti-PGC-1α antibody; Tubulin served as loading control. **(D)** Representative high magnification image of co-staining PGC-1α (Red) with DAPI (Blue). GCL, granule cell layer. **(d)** The proportions of PGC-1α-positive cells in the adult hippocampus are presented by %PGC-1α^+^/DAPI^+^ cells. Values are means ± S.E.M., *n* = 6/group, ***p < 0.001 for Ctr vs. *Pgc-1*α CKO mice.

To more intuitively investigate how PGC-1α regulates hippocampal neuron survival and proliferation, we generated an inducible, hippocampus-specific PGC-1α conditional knockout (CKO) mouse model (Calb1-Cre::*Pgc-1*α^fl/fl^) by crossing Calb1-Cre with Pgc-1α^fl/fl^ mice (Figures 4A, B). Immunofluorescence showed reduced PGC-1α-positive cells in the hippocampus after gene deletion (Figures 4D, d). Western blot confirmed decreased PGC-1α protein levels (Figures 4C, c). These results validate the successful *Pgc-1α-*CKO model establishment.

### 3.5 Pgc-1α gene deletion reduces neuron numbers by inhibiting the FNDC5/BDNF/TrkB pathway

**FIGURE 5:**
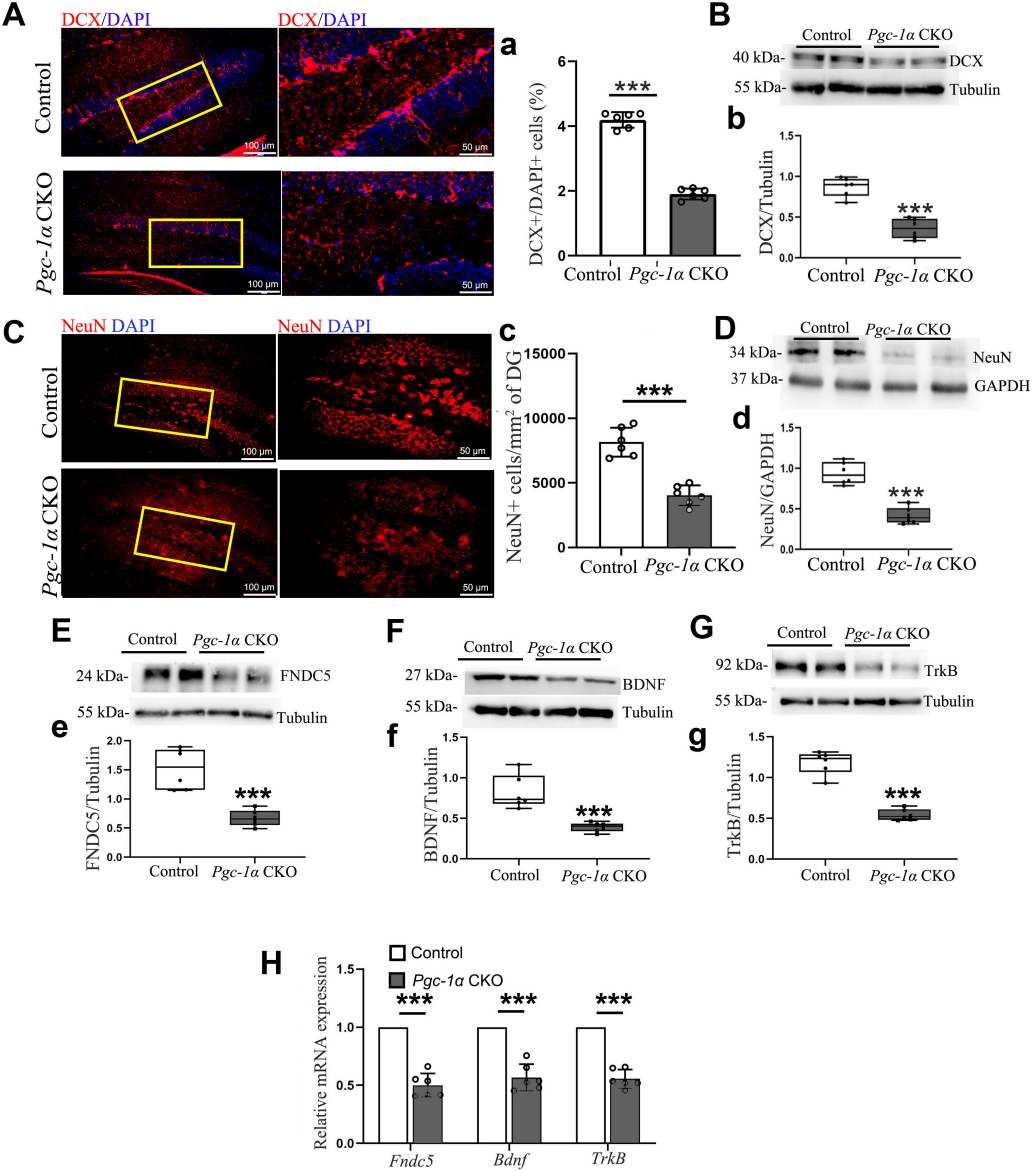
*Pgc-1*α gene deletion reduces immature and mature neuron numbers by inhibiting the FNDC5/BDNF/TrkB pathway. **(A)** DCX and **(C)** NeuN immunolabeling on hippocampal sections from both genotypes. Red, DCX or NeuN. Blue, DAPI. *n* = 6/group. **(a)** The quantification of numbers of DCX-positive cells in AD hippocampus was presented as %DCX^+^/DAPI^+^ cells. **(c)** The average density of neurons expressing NeuN protein per unit area (mm^2^) was quantified in the adult hippocampal DG. Hippocampal lysates were immunoblotted for **(B,b)** DCX, **(D,d)** NeuN, **(E,e)** FNDC5, **(F,f)** BDNF, and **(G,g)** TrkB; Tubulin/GAPDH served as loading controls. **(H)** RT-qPCR assessed mRNA levels of *Fndc5, Bdnf*, and *TrkB* in the hippocampus. Values are means ± S.E.M., *n* = 6/group, and significant levels set at ***p < 0.001 indicating differences between Control- and *Pgc-1*α CKO mice.

To investigate whether the deletion of the *Pgc-1*α gene reduces the formation of both immature and mature neurons, by utilizing immunofluorescence and Western blot analyses, we confirmed that PGC-1α deletion decreased DCX^+^ and NeuN^+^ cell numbers (Figures 5A, a, C, c) and DCX/NeuN protein levels (Figures 5B, b, D, d). Furthermore, Western blot revealed reduced FNDC5, BDNF, and TrkB expression (Figures 5E–G, e–g). RT-qPCR confirmed downregulated *Fndc5*, *Bdnf*, and *TrkB* transcription in *Pgc-1*α CKO hippocampal tissues compared to controls (Figure 5H).

## 4 Discussion

During adult neurogenesis, naturally occurring cell death accounts for more than half of the differentiating neurons in the brain (Kuhn, 2015). Notably, extensive research has established a link between neurogenesis defects and Alzheimer’s disease (AD), with reduced survival of newborn neurons representing one of the most well-documented abnormalities in the AD hippocampus (Pan et al., 2025). A deeper understanding of the survival-promoting factors involved in adult neurogenesis is critical for evaluating this process as a potential therapeutic target for AD.

Our prior research has established that sustained PGC-1α expression is critical for mitigating AD-like cognitive impairments and neuronal apoptosis (Shi et al., 2024; Wang et al., 2021, 2022a). The therapeutic potential of PGC-1α in AD may involve restoring mitochondrial fission-fusion dynamics and normalizing its distribution in AD-affected brains (Shi et al., 2024; Wang et al., 2022a). However, the role of PGC-1α in regulating cell survival during hippocampal neurogenesis in AD, along with its underlying mechanisms, remains largely uncharacterized. This study reveals several key insights: First, we demonstrate that enhancing PGC-1α expression in the hippocampal neurogenic niche improves both short-term and long-term survival of newborn DG neurons in an AD context. Second, our findings indicate that PGC-1α upregulation promotes the differentiation and proliferation of newborn DG neurons within the AD hippocampus. Lastly, we establish that PGC-1α functions as an upstream regulator of FNDC5-BDNF signaling to support DG neuron survival.

In rodents, a significant proportion (30%–80%) of newly generated granule cells in the hippocampus undergo programmed cell death. Those that survive beyond 2 weeks migrate toward the granule cell layer (Toni and Schinder, 2016), initiate axonal and dendritic development, and ultimately integrate into the hippocampal network. Based on this observation, we investigated the effects of PGC-1α on the survival of DCX-labeled immature neurons and NeuN-labeled mature neurons following EdU pulse-labeling of proliferating cells for 2 and 6 weeks. Our findings demonstrate that PGC-1α overexpression enhances the survival of adult-born granule cells within the AD hippocampus.

BDNF plays a critical role in stimulating the differentiation and survival of newly generated neurons during adult hippocampal neurogenesis (Chan et al., 2008). We hypothesize that PGC-1α promotes the survival of fully matured neurons, at least in part, by regulating BDNF signaling. Co-staining of the neurotrophic factor BDNF with NeuN reveals that PGC-1α contributes to the proliferation of BDNF-positive neurons. While the precise mechanisms remain to be fully elucidated, recent studies offer plausible explanations. Specifically, the BDNF-TrkB axis stimulates neuronal differentiation via activation of the PI3K-Akt and MAPK/ERK pathways (Sun et al., 2019; Wang et al., 2024), which upregulate the transcription factor Prox1 to direct neuroblasts toward DCX-positive immature neuron lineages (Obara and Hunt, 2013). Concurrently, BDNF-TrkB-mediated phosphorylation of CREB increases anti-apoptotic Bcl-2 expression, suppressing mitochondrial apoptosis pathways and ensuring the survival of NeuN-positive mature neurons (Soman et al., 2025). Based on these findings, we examined the survival-promoting effects of the BDNF/TrkB axis in this study. Notably, AAV-PGC-1α infusion was found to upregulate both BDNF and TrkB expression in the hippocampus of AD models.

Recent work demonstrates that PGC-1α coactivates estrogen-related receptor alpha (ERRα) in cortical and hippocampal neurons. This interaction regulates *Fndc5* gene transcription, leading to increased BDNF expression (Wrann et al., 2013). In a review summarizing the mechanisms underlying exercise’s effects on neurodegenerative disorders further confirmed the role of exercise in activating the PGC-1α/FNDC5/BDNF signaling pathway (Jodeiri Farshbaf et al., 2016). Based on these findings, we investigated the involvement of PGC-1α in regulating FNDC5 transcription and expression in the hippocampus of AD models. In our study, AAV-PGC-1α infusion into the DG of the hippocampus elevated FNDC5 transcription and expression. Given FNDC5’s positive regulation of BDNF expression, we propose that the survival of fully matured neurons in AD is mediated, at least partially, through the modulation of the PGC-1α-FNDC5-BDNF-TrkB axis.

Interestingly, existing evidence suggests that regular physical exercise enhances BDNF levels, which may subsequently activate the PGC-1α-FNDC5 pathway (Bi et al., 2024). This activation stimulates the mechanistic target of rapamycin (mTOR) signaling, thereby promoting local protein synthesis within axons (Obara and Hunt, 2013). However, a comprehensive understanding of whether PGC-1α or BDNF serves as the upstream regulator for newborn cell survival during AD-associated hippocampal neurogenesis remains unclear. To identify downstream effectors of these pathways, we generated Calb1^+^neuron-*Pgc-1*α-CKO mice by crossing Calb1-Cre mice with those harboring a floxed PGC-1α allele. Calb1, an important calcium-binding protein regulating intracellular Ca^2+^ concentration, is predominantly expressed in brain regions associated with cognition and memory, including the cortex, hippocampus, striatum, and thalamus (Yang et al., 2018). Studies have shown that pathological tau changes selectively affect pyramidal neurons with high Calb1 expression (Datta et al., 2021). The underlying mechanism involves calcium overload, specifically increased intracellular Ca^2+^ concentration, which induces neurotoxicity via tau phosphorylation through multiple synergistic pathways (Datta et al., 2021). This neurotoxicity is exacerbated by the significant loss of the calcium-binding protein Calb1 (Datta et al., 2021). To investigate the effects of Calb-Cre recombination-mediated deletion, we compared the number of PGC-1α-positive cells and PGC-1α expression levels between genotypes. Preliminary studies demonstrated that the Calb promoter induces a specific reduction in PGC-1α-positive neurons within the hippocampus, particularly in the subgranular zone and granule cell layer, which are critical for hippocampal neurogenesis. Additionally, reduced PGC-1α expression was accompanied by markedly inhibited survival of newborn neurons in the hippocampal DG, as evidenced by decreased numbers of DCX-positive immature neurons and NeuN-positive mature neurons. Using this mouse model, we further examined the impact of PGC-1α knockout on BDNF expression. Reduced BDNF expression confirmed that BDNF is a downstream target of PGC-1α. Furthermore, gene expression analysis provided additional evidence that PGC-1α maintains newborn cell survival via the FNDC5-BDNF-TrkB pathway, consistent with our previous conclusion that PGC-1α promotes adult neurogenesis by activating neurotrophic signaling in AD.

## 5 Conclusion

Together, this study provides the first demonstration that PGC-1α regulates the survival of newborn neurons, thereby contributing to adult hippocampal neurogenesis in AD models. Additionally, we elucidate the mechanism through which PGC-1α enhances newborn neuron survival. Our findings indicate that PGC-1α signaling modulates the FNDC5-BDNF-TrkB pathway, and its loss inhibits DG neuron survival while disrupting neurotrophic signaling. Future studies integrating single-cell transcriptomics, lineage tracing, and circuit-specific manipulations will be essential for further dissecting these mechanisms and evaluating the therapeutic potential of targeting this axis in neurodegenerative disorders.

## Data Availability

The original contributions presented in this study are included in this article/Supplementary material, further inquiries can be directed to the corresponding authors.
