# Supplementary material for: PGC-1α promotes the survival of newborn neurons within AD hippocampus through activation of the FNDC5/BDNF/TrkB signaling pathway
Source: Front Mol Neurosci. 2025 Oct 14;18:1688694. doi: 10.3389/fnmol.2025.1688694 (PMC12558988; doi:10.3389/fnmol.2025.1688694)
Supplement: Supplementary file 1 [file Table_1.docx]

|  | Group | **Shapiro-Wilk test** | | |
| --- | --- | --- | --- | --- |
|  |  | W | n | p value |
| Figure2 b1  DCX+EDU+/DAPI+ cells (%) | APP/PS1+  AAV-Control | 0.883 | 6 | 0.281 |
|  | APP/PS1+  AAV-PGC-1α | 0.946 | 6 | 0.707 |
| Figure2 b2  DCX+ cells/mm2 of DG | APP/PS1+  AAV-Control | 0.897 | 6 | 0.355 |
|  | APP/PS1+  AAV-PGC-1α | 0.947 | 6 | 0.718 |
| Figure2 d1  NeuN+EDU+/DAPI+ cells (%) | APP/PS1+  AAV-Control | 0.907 | 6 | 0.415 |
|  | APP/PS1+  AAV-PGC-1α | 0.859 | 6 | 0.187 |
| Figure2 d2  NeuN+ cells/mm2 of DG | APP/PS1+  AAV-Control | 0.902 | 6 | 0.383 |
|  | APP/PS1+  AAV-PGC-1α | 0.949 | 6 | 0.736 |
| Figure3 a1  %BDNF+NeuN+/DAPI+ cells | APP/PS1+  AAV-Control | 0.885 | 6 | 0.293 |
|  | APP/PS1+  AAV-PGC-1α | 0.947 | 6 | 0.714 |
| Figure3 a2  %BDNF+NeuN+/NeuN+ cells | APP/PS1+  AAV-Control | 0.920 | 6 | 0.506 |
|  | APP/PS1+  AAV-PGC-1α | 0.853 | 6 | 0.165 |
| Figure3 b  FNDC5/Tubulin | APP/PS1+  AAV-Control | 0.949 | 6 | 0.732 |
|  | APP/PS1+  AAV-PGC-1α | 0.885 | 6 | 0.294 |
| Figure3 c  BDNF/Tubulin | APP/PS1+  AAV-Control | 0.965 | 6 | 0.854 |
|  | APP/PS1+  AAV-PGC-1α | 0.850 | 6 | 0.156 |
| Figure3 d  TrkB/Tubulin | APP/PS1+  AAV-Control | 0.907 | 6 | 0.420 |
|  | APP/PS1+  AAV-PGC-1α | 0.928 | 6 | 0.568 |
| Figure3 e  FNDC5 (RT-qPCR) | APP/PS1+  AAV-Control | . | 6 | . |
|  | APP/PS1+  AAV-PGC-1α | 0.882 | 6 | 0.279 |
| Figure3 e  BDNF (RT-qPCR) | APP/PS1+  AAV-Control | . | 6 | . |
|  | APP/PS1+  AAV-PGC-1α | 0.900 | 6 | 0.377 |
| Figure3 e  TrkB (RT-qPCR) | APP/PS1+  AAV-Control | . | 6 | . |
|  | APP/PS1+  AAV-PGC-1α | 0.957 | 6 | 0.793 |
| Figure3 f1  Nrs.of BDNF+NeuN+ cells/DAPI+ cells | APPSwe+pEnCMV | 0.941 | 6 | 0.664 |
|  | APPSwe+*Pgc-1α* | 0.943 | 6 | 0.682 |
| Figure3 f2  %BDNF+NeuN+/NeuN+ cells | APPSwe+pEnCMV | 0.950 | 6 | 0.742 |
|  | APPSwe+*Pgc-1α* | 0.894 | 6 | 0.342 |
| Figure3 h  FNDC5/Tubulin | APPSwe+pEnCMV | 0.880 | 6 | 0.270 |
|  | APPSwe+*Pgc-1α* | 0.928 | 6 | 0.563 |
| Figure3 i  BDNF/Tubulin | APPSwe+pEnCMV | 0.923 | 6 | 0.529 |
|  | APPSwe+*Pgc-1α* | 0.883 | 6 | 0.283 |
| Figure3 j  TrkB/Tubulin | APPSwe+pEnCMV | 0.856 | 6 | 0.177 |
|  | APPSwe+*Pgc-1α* | 0.943 | 6 | 0.681 |
| Figure4 c  PGC-1α/Tubulin | Control | 0.946 | 6 | 0.711 |
|  | *Pgc-1α* CKO | 0.971 | 6 | 0.902 |
| Figure4 d  PGC-1α+/DAPI+ cells (%) | Control | 0.936 | 6 | 0.624 |
|  | *Pgc-1α* CKO | 0.881 | 6 | 0.273 |
| Figure5 a  DCX+/DAPI+ cells (%) | Control | 0.955 | 6 | 0.784 |
|  | *Pgc-1α* CKO | 0.841 | 6 | 0.133 |
| Figure5 b  DCX/Tubulin | Control | 0.916 | 6 | 0.476 |
|  | *Pgc-1α* CKO | 0.915 | 6 | 0.472 |
| Figure5 c  NeuN+ cells/mm2 of DG | Control | 0.898 | 6 | 0.364 |
|  | *Pgc-1α* CKO | 0.923 | 6 | 0.528 |
| Figure5 d  NeuN/GAPDH | Control | 0.906 | 6 | 0.412 |
|  | *Pgc-1α* CKO | 0.917 | 6 | 0.487 |
| Figure5 e  FNDC5/Tubulin | Control | 0.821 | 6 | 0.091 |
|  | *Pgc-1α* CKO | 0.992 | 6 | 0.993 |
| Figure5 f  BDNF/Tubulin | Control | 0.867 | 6 | 0.216 |
|  | *Pgc-1α* CKO | 0.964 | 6 | 0.850 |
| Figure5 g  TrkB/Tubulin | Control | 0.866 | 6 | 0.212 |
|  | *Pgc-1α* CKO | 0.919 | 6 | 0.496 |
| Figure5 h  FDNC5 (RT-qPCR) | Control | . | 6 | . |
|  | *Pgc-1α* CKO | 0.879 | 6 | 0.265 |
| Figure5 h  BDNF (RT-qPCR) | Control | . | 6 | . |
|  | *Pgc-1α* CKO | 0.892 | 6 | 0.331 |
| Figure5 h  TrkB (RT-qPCR) | Control | . | 6 | . |
|  | *Pgc-1α* CKO | 0.889 | 6 | 0.311 |
